# Scattering and Gaussian Fluctuation Theory for Semiflexible Polymers

**DOI:** 10.3390/polym8090301

**Published:** 2016-09-13

**Authors:** Xiangyu Bu, Xinghua Zhang

**Affiliations:** School of Science, Beijing Jiaotong University, Beijing 100044, China; 12272030@bjtu.edu.cn

**Keywords:** worm-like chain model, structure factor, Gaussian fluctuation theory, random phase approximation

## Abstract

The worm-like chain is one of the best theoretical models of the semiflexible polymer. The structure factor, which can be obtained by scattering experiment, characterizes the density correlation in different length scales. In the present review, the numerical method to compute the static structure factor of the worm-like chain model and its general properties are demonstrated. Especially, the chain length and persistence length involved multi-scale nature of the worm-like chain model are well discussed. Using the numerical structure factor, Gaussian fluctuation theory of the worm-like chain model can be developed, which is a powerful tool to analyze the structure stability and to predict the spinodal line of the system. The microphase separation of the worm-like diblock copolymer is considered as an example to demonstrate the usage of Gaussian fluctuation theory.

## 1. Introduction

In most theoretical research of polymers, the Gaussian chain (GSC) model has been employed to describe the chain configuration of polymers. The GSC model utilizes a harmonic potential for the intra-chain bonding interaction. It facilitates analytical and numerical calculations. However, a number of unphysical properties arise simultaneously when it is subject to strong external fields [1]. It is a coarse-grained model and only adequate for the flexible polymer on the level of the mesoscopic scale comparable to the radius of gyration, $R_{g}$. For example, the short block copolymers have drawn considerable attention recently because of their exciting new technological applications, such as block copolymer lithography [2]. Besides, surfactants and lipids modeled as short amphiphilic molecules from the view of coarse graining are being intensely researched, both experimentally and theoretically for nano-technological and biological applications [3,4,5].

The above limitations primarily can be circumvented by introducing the semiflexible chain model, such as the freely-jointed chain model [6], the rigid Gaussian chain model [7], the discrete [8] and the continuous worm-like chain model [9]. Among these models, the worm-like chain (WLC) model, where the polymer chain is theoretically treated as an inextensible thread with a fixed contour length *L* and appears rigid approximately within a segment of a persistence length $l_{p}$. Another advantage of the WLC model is the additional orientational dependence. This is essential for dealing with the spatially-inhomogeneous polymer liquid-crystal systems [10,11,12,13]. Moreover, the WLC model is a multi-scale chain model. It can comprehensively describe the structural properties from the microscopic persistence length to the mesoscopic chain length. In the experiment, the light- and neutron-scattering techniques are employed to probe the density-density correlation of the polymer chain in different length scales. The scattering pattern measured by the experiment is the structure factor of the target system. In theory, the structure factor S(k) is the Fourier transformation of the density-density correlation function, i.e.,

(1) $$\begin{array}{c} S\left(k\right)\equiv \int d\Delta rf\left(\Delta r\right)\exp\left(ik\cdot \Delta r\right) \end{array}$$

In real space, the density-density correlation function of a homogeneous system is defined by [14]:

(2) $$f\left(\Delta r\right)\equiv \langle \hat{\phi }\left(r\right)\hat{\phi }\left(r^{\prime }\right)\rangle$$

where the $\langle ...\rangle$ is the ensemble average and $\Delta r=r-r^{\prime }$. The monomer density operator for a given configuration, R(s), of the chain in space is defined by:

(3) $$\hat{\phi }\left(r\right)=\frac{L}{a\rho_{0}}\int_{0}^{1}ds\delta \left[R\left(s\right)-r\right]$$

where the average number density of monomers is given by $\rho_{0}=nL/\left(aV\right)$, *n* is the chain number and *V* is the volume of the system.

Many fundamental physical properties of polymers, such as molecular weight, the radius of gyration, persistent length and the density–density correlation of the polymeric systems [15], can be determined by fitting scattering data with a specific microscopic chain model. The theoretical prediction of the structure factor from the microscopic chain models can be traced back to the early development of polymer physics [16]. Therefore, the structure factor serves as a bridge connecting the theory and experiment. One example is the structure factor of the GSC model, which is widely used to study the small-angle scattering behaviors of the polymer chain with high a degree of polymerization. It takes a well-known Debye function form [17],

(4) $$S_{\mathrm{Debye}}\left(x\right)=\frac{2}{x^{2}}\left[\exp\left(-x\right)-1+x\right]$$

where $x=k^{2}R_{g}^{2}$ and $R_{g}$ is the radius of gyration. Moreover, the bond correlation effect becomes important for the semiflexible [18] and rigid chain [19]. From the structure factor, the size and architectures (linear, ring or dendrimer) of the polymers can be studied by the scattering experiment [20]. Furthermore, predicting the property of material from its structure is one of the key problems the in material science. As defined in Equation (1), the structure factor is the Fourier transformation of the second order moment of the density operator. The first order moment of the density operator describes the structure of the system; while the second order moment of the density operator characterizes the linear response of the structure to the external stimulation in different length scales. Using the random phase approximation (RPA), the structure factor of an ordered structure can be obtained based on the structure factor of a single chain in an external field as an input [21]. The intrinsic eigenvalues of the structure factor are the linear elastic moduli of the structure, which depend on the composition and interaction parameter. The spinodal boundary is the condition that makes one of the moduli diminish, and then, the structure becomes unstable. This mode is called the soft mode of the structure, and its corresponding eigenvector indicates the evaluating path of the unstable structure [14]. The successful determination of the spinodal curve for the formation of micro-domains in diblock copolymers is an excellent example of using the structural factor [22]; the theory predicts the instability of the homogenous state against inhomogeneous density perturbations that lead to the formation of a microscopic structure based on the GSC model. The spinodal line of the ordered system can be determined by the same theory. In the inhomogeneous system, the fluctuation become anisotropic, and the structure factor of the ordered structure is needed; for example, the anisotropic fluctuation effects of the self-assembly structure of the diblock copolymer [23].

Incorporating the renormalization technology, the fluctuation effect can be investigated [24]. Moreover, the structure factor is also the starting point of the polymer reference interaction site model (RISM) theory from which the close packing structure in the microscopic scale can be obtained [25]. The local structure is extremely crucial to understand the correlation effects in many important polymeric systems, such as the polymer adsorption [26,27] and the polyelectrolytes [28,29,30]. In addition, the correlation of the structure in different length scales is critical for the study of the dynamic properties of polymeric systems far from equilibrium, such as the glass transition [31] and the diffusion of nano-particles in polymer matrices [32].

There is, however, a large class of semiflexible polymer chains, where the effects of rigidity are important, which cannot be described by the Gaussian chain model. In the WLC model proposed by Saito, Takahashi and Yunoki [9], which is a continuous version of the Kratky–Porod model, a smooth spacial curve R(s), where *s* is an arc-variable continuously varying from one end (s=0) to another (s=1) of total length *L*, is used to describe the conformation of the polymer chain [9,17,33]. An energy penalty relating to the local curvature of the curve is introduced to describe the rigidity of the chain. The Boltzmann weight for such a configuration is then given by:

(5) $$W\left[R\left(s\right)\right]=\exp\left[-\beta H_{0}\right]$$

where $\beta =1/k_{B}T$, *T* is the temperature and $k_{B}$ the constant. The Hamiltonian:

(6) $$\beta H_{0}=\frac{a}{4L}\int_{0}^{1}ds|\frac{dt(s)}{ds}{|}^{2}+\frac{L}{a}\int_{0}^{1}dsw[R\left(s\right),t\left(s\right)]$$

In a general case, the polymer chain is subject to an external field, w[R(s),t(s)] [34]. The tangent vector t(s)≡(1/L)dR(s)/ds specifies the local orientation of the polymer chain at location *s* and makes sure the curve is smooth. Moreover, the local inextensible condition is introduced by the constraint of |t(s)|=1. For comparison with the results of a Gaussian-chain model, the persistence length $l_{p}$ is replaced by Kuhn length *a* by considering:

(7) $$a=2l_{p}$$

in this review. Then, the WLC model involves two characteristic length scales: the length of chain *L* and the effective Kuhn length *a*.

The statistical properties of a WLC can be fully described by the propagator $g(r-r^{\prime };t^{\prime },t;s)$. It is the joint probability of finding a polymer chain section of contour length *s* along the chain, in such a condition that the head is located at a position represented by the coordinate vector $r^{\prime }$ and points to a direction specified by a unit vector $t^{\prime }$, as well as that the tail is located at a position represented by the coordinate vector r and points to a direction specified by a unit vector t. Once a homogeneous system with translational symmetry is considered, the propagator *g* only depends on $\Delta r\equiv r-r^{\prime }$.

Following the standard treatment, one can show that the calculation of the Green’s function in three dimensions can proceed by solving the modified diffusion equation (MDE) [33]:

(8) $$\begin{array}{c} \frac{\partial }{\partial s}g\left(r,r^{\prime };t^{\prime },t;s\right)=\left[\frac{L}{a}\nabla_{t}^{2}-Lt\cdot \nabla_{r}+w\left(r,t\right)\right]g\left(r,r^{\prime };t^{\prime },t;s\right) \end{array}$$

which is subject to the initial condition $g\left(r,r^{\prime };t^{\prime },t;0\right)=\delta \left(r-r^{\prime }\right)\delta \left(t-t^{\prime }\right)$. The ensemble average of any observable can be computed from the propagator. Then, the density-density correlation function can be expressed as [14]:

(9) $$\begin{array}{ccc} \langle f\left(r-r^{\prime }\right)\rangle & = & \frac{1}{{\left(4\pi \right)}^{4}Q}\int_{0}^{1}ds\int_{0}^{s}ds^{\prime }\int dt_{0}dt^{\prime }dtdt_{1}\int dr_{0}dr_{1}\\ & \times & g\left(r_{0}-r^{\prime };t_{0},t^{\prime };s^{\prime }\right)g\left(r^{\prime }-r;t^{\prime },t;s-s^{\prime }\right)g\left(r-r_{1};t,t_{1};1-s\right)\\ & + & \frac{1}{{\left(4\pi \right)}^{4}Q}\int_{0}^{1}ds\int_{0}^{s}ds^{\prime }\int dt_{0}dt^{\prime }dtdt_{1}\int dr_{0}dr_{1}\\ & \times & g\left(r_{0}-r;t_{0},t;s^{\prime }\right)g\left(r-r^{\prime };t,t^{\prime };s-s^{\prime }\right)g\left(r^{\prime }-r_{1};t^{\prime },t_{1};1-s\right) \end{array}$$

where *Q* is the single-chain partition function. Then, the structure factor can be obtained by performing the Fourier transformation. For a general formation of w(r,t), it is difficult to obtain the full propagator, $g(\Delta r;t^{\prime },t;s)$ even through the numerical method due to its high dimensional dependence. In this review, we consider the spacial homogeneous system where the translational symmetry is satisfied. Namely, the field *w* only depends on t.

In this review: (a) the multi-scale behaviors of the WLC model are discussed by analyzing the scattering function of the ideal WLC in Section 2; (b) the anisotropic behaviors of a single WLC in a nematic field are shown in Section 3; (c) the spinodal lines of the self-assembly of the semiflexible diblock copolymer are demonstrated in Section 4 as an example of the application of the structure factor in Gaussian fluctuation theory; moreover, (d) the multi-scale nature of the WLC model microscopic interaction range effects is discussed in Section 4.3.

## 2. Structure Factor of a Single Worm-Like Chain (WLC)

For the condition of an ideal chain in a spatially-homogenous and directionally-disordered system, in which the external field w=0, the end integral of the propagator can be obtained analytically,

(10) $$\frac{1}{4\pi }\int dt_{0}\int dr_{0}g\left(r_{0}-r;t_{0},t;s\right)=1$$

and

(11) $$\frac{1}{4\pi }\int dt_{1}\int dr_{1}g\left(r-r_{1};t,t_{1};1-s\right)=1$$

for any r, t and *s*, as well as Q=1. Thus, the density-density correlation function can be simplified as:

(12) $$\begin{array}{ccc} \langle f\left(r-r^{\prime }\right)\rangle & = & \frac{1}{{\left(4\pi \right)}^{2}}\int_{0}^{1}ds\int_{0}^{s}ds^{\prime }\int dt\int dt^{\prime }[g\left(r-r^{\prime },t,t^{\prime };s-s^{\prime }\right)\\ & & \;\;\;\;\;\;+g(r^{\prime }-r,t^{\prime },t;s-s^{\prime })] \end{array}$$

Additionally, then the structural factor can be accessed,

(13) $$\begin{array}{ccc} S(ka;L/a) & \equiv & \int d\Delta rf\left(\Delta r\right)\exp\left[-ik\cdot \Delta r\right]\\ & = & \frac{1}{4\pi }\int_{0}^{1}ds\int_{0}^{s}ds^{\prime }\int dt[G\left(k,t,s-s^{\prime }\right)+G\left(-k;t;s-s^{\prime }\right)] \end{array}$$

Here, the end-integral propagator in Fourier space is defined as:

(14) $$G\left(k,t;s\right)\equiv \int dt^{\prime }\left[\int d\Delta rg\left(\Delta r;t^{\prime },t;s\right)\exp\left(ik\cdot \Delta r\right)\right]$$

which satisfies:

(15) $$\begin{array}{c} \frac{\partial }{\partial s}G\left(k,t,s\right)=\left[\frac{L}{a}\nabla_{t}^{2}+iLk\cdot t\right]G\left(k,t,s\right) \end{array}$$

with the initial condition of G(k,t,0)=1. Choose the direction of wave vector k as the *z* axis. The MDE becomes:

(16) $$\begin{array}{c} \frac{\partial }{\partial s}G\left(k,\theta ;s\right)=\frac{L}{a}\frac{1}{\sin\theta }\frac{\partial }{\partial \theta }\left[\sin\theta \frac{\partial G(k,\theta ,s)}{\partial \theta }\right]+iLk\cos\theta G\left(k,\theta ,s\right) \end{array}$$

and the structure factor only depends on wavenumber *k*:

(17) $$\begin{array}{c} S\left(ka;L/a\right)=\frac{1}{2}\int_{0}^{1}ds\int_{0}^{s}ds^{\prime }\int d\cos\theta [G\left(k,\theta ,s-s^{\prime }\right)+G\left(-k,\theta ,s-s^{\prime }\right)] \end{array}$$

In summary, to calculate S(ka;L/a), first solve the MDE, Equation (16), for given wavenumber *k* and L/a; then perform the integration according to Equation (17).

### 2.1. Rigid and Flexible Limit

An advantage of the WLC model is that it can describe the structural properties of the entire range of flexibility, L/a. the Gaussian-chain and rigid-rod-chain structural factor can be analytically recovered from the current model, at the limit of L/a≫1 and L/a≪1, respectively. To recover the Gaussian chain model in the flexible chain limit, L/a≫1, expand G(k,θ,s) in terms of Legendre functions $P_{l}\left(\cos\theta \right)$,

(18) $$G\left(k,\theta ,s\right)=\sum_{l\;=\;0}^{\infty }\gamma_{l}\left(ka,s\right)P_{l}\left(\cos\theta \right)$$

Substituting this expression into Equation (16), a set of differential equations for $\gamma_{l}\left(ka,s\right)$ is obtained. For l=0,

(19) $$\frac{\partial }{\partial s}\gamma_{0}\left(ka,s\right)=\left(ika\right)\frac{1}{3}\frac{L}{a}\gamma_{1}\left(ka,s\right)$$

For l=1,2,3,⋯,

(20) $$\begin{array}{ccc} \frac{\partial }{\partial s}\gamma_{l}\left(ka,s\right) & = & -\frac{L}{a}l\left(l+1\right)\gamma_{l}\left(ka,s\right)+\left(ika\right)\frac{L}{a}\frac{l}{2l-1}\gamma_{l\;-\;1}\left(k,s\right)\\ & & +\left(ika\right)\frac{L}{a}\frac{l+1}{2l+3}\gamma_{l\;+\;1}\left(ka,s\right) \end{array}$$

In the flexible chain limit, the anisotropic components of the propagator should decay as quickly as possible, which means $\frac{\partial }{\partial s}\gamma_{l}\left(ka,s\right)=0$ for l≠0. According to Equation (20), this requires $ka∼\sqrt{a/L}$. This means the orientational bias from the chain rigidity can be ignored on the length scale, which is larger than *a*. In this regime, the isotropic component of the propagator satisfies:

(21) $$\frac{\partial }{\partial s}\gamma_{0}\left(ka,s\right)=-\frac{{\left(ka\right)}^{2}}{6}\frac{L}{a}\gamma_{0}\left(ka,s\right)+O{\left(ka\right)}^{2}$$

which is identical to the *k*-space representation of MDE for a GSC model. Its solution is $g\left(k,s\right)=\exp[-{\left(kR_{g}\right)}^{2}s]$. Using the definition of structure factor Equation (17), one can obtain the Debye function as shown in Equation (4).

The solution of Equation (16) at the rigid limit (L/a≪1) was first determined by Neugebauer in 1943 [35]. Within this limit, the first term on the right-hand side of Equation (16) disappears. An analytical solution to this equation can be found:

(22) G(ka,θ,s)=exp(isLkcosθ)

Following the definition in Equation (17), the structure factor has the following expression,

(23) $$\begin{array}{c} S_{\mathrm{rod}}\left(kL\right)=\frac{2}{{\left(kL\right)}^{2}}\left[-1+kL\int_{0}^{kL}\frac{\sin y}{y}dy+\cos\left(kL\right)\right] \end{array}$$

where the parameter *a* completely disappears from the expression on the right-hand side and the main length scale in the problem is *L*. Thus, in the rigid regime (L/a≤1), it makes sense to plot *S* directly as a function of kL.

### 2.2. Asymptotic Behavior of the Structure Factor in Small and Large Wavenumbers

The light- and neutron-scattering techniques are usually employed to probe the structural factor experimentally. The density-density correlation of the polymer chain in the different length scales can be accessed from different *k* regimes in the scattering pattern. The low *k* region of the structure factor corresponds to the signal of small-angle-scattering, which characterizes the overall structure of the polymer chain, and the large *k* region of the structure factor corresponds to the wide-angle-scattering, which characterizes the local structure. The structure factor satisfies $S∼k^{-d}$, where *d* is the fractal dimension of the structure. Because the polymer chain is a multi-scale system, in different length scale regions, *d* has a different value. The boundaries between these regions define the characteristic length scales of the polymer. There are two characteristic length scales, the radius of gyration, $R_{g}$, and the Kuhn length, *a*. Consider the ideal flexible chain as an example, i.e., L/a≫1. For $k\ll 2\pi /R_{g}$, the length scale is sufficiently large that the chain can be considered as a point with d=0. For $2\pi /a<k<2\pi /R_{g}$, the center limit theorem requires that the conformation of the chain is a random walk. The fractal dimensions of the random walk are two. For k≫2π/a, the orientation of adjacent bonds along the chain is highly correlated. The linear shape of the chain indicates its fractal dimension is 1. In a double-logarithmic coordinate, the scattering data are a polygonal line with different slopes in different wavenumber regions. In practice, Kratky function, which is defined as $k^{2}S\left(k\right)$, is used to analyze the structure of the chain. In double-logarithmic coordinates, the slope of the Kratky function is 2−d. Then the two-dimensional structure, which characterizes the random walk behavior, is a platform. Therefore, it is convenient to check whether the conformation of the chain satisfies the behavior of the random walk or not.

In the low *k* regime (commonly called the Guinier regime), the spatial resolution approaches the overall size of the polymer chain. The global conformational information of the chain can be probed. In this regime, the structure factor can be approximated by:

(24) $$S\left(ka\ll 1,L/a\right)=1-{\left(Lk\right)}^{2}\langle R_{g}^{2}\rangle /3L^{2}+...$$

where $\langle R_{g}^{2}\rangle$ is the radius of gyration. $\langle R_{g}^{2}\rangle$ can be measured by the small angle scattering experiment. The radius of gyration of the discrete non-Gaussian chain model including (1) the constant valence angle chain with free rotation, (2) the discrete WLC and (3) the constant valence angle chain with restricted rotation were predicted by Benoit and Doty in the 1950s [18].

(25) $$\langle R_{g}^{2}\rangle =\frac{aL}{6}\left[1-\frac{3a}{2L}+\frac{3a^{2}}{2L^{2}}-\frac{3a^{3}}{4L^{3}}\left(1-e^{-2L/a}\right)\right]$$

is the exact expression of the radius of gyration of a WLC [36]. For the flexible limit L/a>>1, $<R_{g}^{2}>=aL/6$, which is consistent with the prediction of Gaussian chain model. According to Equation (24), the structure factor is proportional to $k^{2}$ in the low *k* region. In experiments, this relation is used to predict $R_{g}$ from scattering data.

The structural factor in the large-ka regime for any L/a can be analytically deducted from Equation (16). Taking the ka≫1 limit, we can drop the first term on the right-hand side. Formally, the solution can be connected with the rigid rod limit, because the mathematical formation of the MDE is exactly the same. At the ka≫1 limit, $S∼k^{-1}$:

(26) $$\begin{array}{c} S\left(ka\gg 1\right)=\frac{2}{kL}\int_{0}^{\infty }\frac{\sin y}{y}dy+... \end{array}$$

Hence in the large *k* region of the Kratky plot,

(27) $$\left(L/a\right){\left(ka\right)}^{2}S=\pi ka+...$$

is a straight line that all data should merge together.

**Figure 1:**
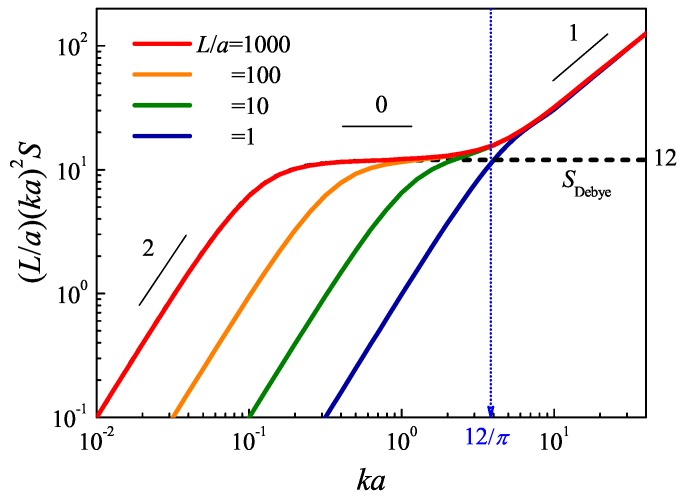
Structure factor of the worm-like chain (WLC) for various L/a in a double-logarithmic Kratky plot. Black solid lines with slopes of 2, 0 and 1 indicate the scaling behavior of the structure factor in different wavenumber regions. The dashed line is the $S_{\mathrm{Debye}}$, and dots indicate the crossover region between random walk behavior in the mesoscopic scale and the linear connection behavior in the microscopic scale.

It should be emphasized that the GSC model is a coarse-grained model, which ignores all of the information in the large *k* regime. The large *k* limit of its the structure factor of Equation (4), $S_{\mathrm{Debye}}\left(x\right)=2/x$. It is a plateau ${\left(ka\right)}^{2}S=12/\left(L/a\right)$ (ka≫1) in the Kratky plot (black dash in Figure 1), which can also be used to predict $R_{g}$ from the scattering data. This behavior indicates in the GSC model that the chain has a self-similar random walk structure, and its fractal dimension is two. This approximation fails to describe local behavior on the length scales close to and below *a*. The WLC model, on the other hand, gives rise to the correct physics and predicts another crossover to a slope of one in the Kratky plot. The intersection of this asymptotic behavior with the GSC behavior $\left(L/a\right){\left(ka\right)}^{2}S_{\mathrm{Debye}}=12$.

(28) $$k^{*}a=12/\pi ,$$

which is indicated in Figure 1 by blue arrow is customarily considered the location that can be used to define a/2, or the persistence length, from experimental measurements [37]. The crossovers between these three regimes have been observed in both experiment [38] and computer simulation [39,40,41,42].

### 2.3. Structure Factor of WLC with Finite L/a

Although there has been a long standing interest in the theoretical calculation of S(ka;L/a) for a WLC, analytical solutions can only be found in the limiting cases L/a≫1 (flexible chain) and L/a≪1 (rigid rod). Previous attempts were made by solving models that were modified from the statistical weight in Equation (6) [43,44,45,46,47]. Kholodenko took the approach of directly approximating the Green’s function, exploiting the similarity between the Green’s function of the semiflexible polymer model and the propagator of Dirac’s fermion, in rigid and flexible limits. Using an interpolation method that matches the two asymptotic limits, he proposed the following analytic formula for the structure factor.

(29) $$S\left(ka;L/a\right)=\frac{2}{y}\left[I_{\left(1\right)}\left(y\right)-\frac{1}{y}I_{\left(2\right)}\left(y\right)\right],$$

where:

(30) $$I_{\left(n\right)}\left(y\right)=\frac{1}{E}\int_{0}^{y}\frac{\sinh(Ez)}{\sinh(z)}z^{n-1}dz,$$

y=3L/a and $E={\left\{\zeta [1-{\left(ak/3\right)}^{2}]\right\}}^{1/2}$, and

(31) $$\zeta =\left\{\begin{array}{cc} 1 & ka\leq 3\\ -1 & ka>3 \end{array}\right.$$

His proposal is by far the simplest, comparing to the approximations proposed earlier by Yoshizaki and Yamakawa [48] and later by Pedersen and Schurtenberger [49]. The Kholodenko formula is an exact solution of Dirac’s fermion problem; however, it is not the solution of the original WLC model in Equation (2). It has been adopted for data-fitting purposes by computational tools, such as FISH [50] and SASFIT [51].

As an effective tool, Monte Carlo simulations can be performed on a discretized version of the WLC model. Pedersen and Schurtenberger performed a series of Monte Carlo simulations of such a chain, with and without the excluded-volume interaction. The structural factor can then be obtained numerically from the simulations [49]. They have provided an empirical formula to represent their simulation data.

(32) $$S\left(ka;L/a\right)=S_{\mathrm{SB}}P+S_{\mathrm{loc}}\left(1-P\right)$$

where:

(33) $$S_{\mathrm{SB}}=S_{\mathrm{Debye}}+b_{2}\frac{a}{L}\left[\frac{4}{15}+\frac{7}{15x}-\left(\frac{11}{15}+\frac{7}{15x}\exp\left(-x\right)\right)\right]$$

(34) $$S_{\mathrm{loc}}=\frac{b_{1}}{Lak^{2}}+\frac{\pi }{Lk}$$

and the empirical weight function has the form of:

(35) $$P=\exp\left[-{\left(\frac{ka}{P_{2}}\right)}^{P_{1}}\right]$$

To obtain good fitting on both conditions of the flexible and rigid chains, the parameters used in Pedersen’s expression depend on L/a (listed in Table 1). $R_{g}$ takes the general expression Equation (25) for L/a≤2 and La/6 for L/a>2, respectively.

The MDE of the WLC model Equation (16) describes the diffusion of a particle on the surface of a unit sphere with a bias external field ikcosθ. Then, the propagator can be expressed in terms of the moment expansion by considering the state without the external field as a reference. In the Fourier–Laplace space, the (2n+1)-th order moments vanish, and the 2n-th order moments can be computed using the so-called stone-fence diagram method [52]. Thus, finding these moments is equivalent to solving the one-dimensional random walk problem for 2n steps with both ends fixed at the same point. Stepanow and coworkers calculated this problem using the same method for computing the dimensions of the irreducible representation of the Temperley–Lieb algebra [53,54]. Numerically, they must invert a matrix of the rank equivalent to the order of the truncated moment in the expansion. A small number of leading moments is adequate for the prediction of the structural factor in the small *k* region. For large the *k* region, more moments are required for a more precise calculation.

A direct calculation of *S* for the standard WLC model, expressed in Equation (6), was not made until 2004 when Spakowitz and Wang provided a semi-analytic approach in the Fourier–Laplace space. They used the constrained one-dimensional random walk to express the propagator of a WLC exactly [55]. They re-grouped the random walk trajectories according to the number of loops. Based on this consideration, the moment expansion can be expressed as an infinite continued fraction. According to Fermat’s last theorem, the calculation of the continued fraction problem is equivalent to inverting a matrix. In fact, the matrix expression of Spakowitz–Wang’s continued fraction has the same format as the matrix used in Stepanow’s work. In order to find the structural factor, however, one must go back to the numerical treatment of the formalism; in particular, an inverse numerical Laplace transformation is needed [55,56]. Besides the linear shape WLC, the structure factor of worm-like rings is studied by both the Monte Carlo simulation and the Daniels approximation [57]. A characteristic peak appears around $R_{g}k$ in the Kratky plot originating from the topological constraint of the ring.

A pseudo-spectral method to obtain G(k,t,s), which solves the MDE Equation (16) directly, is developed to compute the structure factor. S(ka,L/a) [58]. In this method, the propagator is updated by the algorithm of:

(36) $$G\left(k,\theta ,s+\Delta s\right)=e^{i\Delta sLk\cos\theta /2}{\hat{L}}^{-1}\{e^{-\Delta sl(l+1)(L/a)}\hat{L}[e^{i\Delta sLk\cos\theta /2}G\left(k,\theta ,s\right)]\}$$

Here, $\hat{L}$ and ${\hat{L}}^{-1}$ are the operators of Legendre transformation and the Legendre synthesis, respectively. Comparing to the Crank–Nicolson method with a similar degree of complexity [59], this approach is more efficient and easier to manage.

The resulting structure factors of different L/a computed by the above method are compared for the entire wavenumber ka range in Figure 1 [58]. There is an excellent agreement between the two exact numerical solutions by the infinite continued fraction method and the pseudo-spectral method. The approximate analytical forms of the structural factor proposed by Kholodenko and Pedersen works well in both the small and large ka regime. However, in the intermediate ka regime, both methods show deviations, and their deviations depend on chain rigidity, L/a. Kholodenko’s expression has good behavior for both small and large L/a limits, but not in the semiflexible condition, L/a∼1. Pedersen’s expression works well in the large L/a regime, but not in the low L/a regime.

A more specific chain model is the helical WLC model, which includes the energy penalty of the twist conformation [52]. It is a better theoretical model for the biopolymers. However, the extra degree of freedom increases the difficulty for the numerical algorithm. Only the asymptotic method has been used to obtain the structure factor [60].

## 3. Structure Factor in the Orientational Field

The polymer chain can be elongated and orientated by the nematic solution. In the experiment, DNA, neurofilaments and F-actin dispersed in the nematic phase of rod-like fd virus can experience a coil-rod transition [34]. At the mean-field level, the interaction between the target chain and the liquid-crystalline matrix can be studied by an isolated chain in an orientational field obtained from self-consistent field theory. In the Maier–Saupe anisotropic interaction model [61,62], this effective field has a quadrupolar formation $w\left(t\right)=\Gamma \left(t_{z}^{2}-\frac{1}{3}\right)$. Here, the major axis of the nematic phase has been chosen as the *z* axis. The propagator of an isolated WLC in a nematic field satisfies the MDE:

(37) $$\frac{\partial }{\partial s}G\left(k;t^{\prime },t;s\right)=\left[\frac{L}{a}\nabla_{t}^{2}+iLt\cdot k+\Gamma \left(t_{z}^{2}-\frac{1}{3}\right)\right]G\left(k;t^{\prime },t;s\right)$$

which is subject to the initial condition $G\left(k;t^{\prime },t;s=0\right)=\delta \left(t-t^{\prime }\right)$.

By introducing the end integral propagators:

(38) $$\frac{1}{4\pi }\int dt_{0}\int dr_{0}g\left(r_{0}-r;t_{0},t;s\right)=q\left(t,s\right)$$

and:

(39) $$\frac{1}{4\pi }\int dt_{1}\int dr_{1}g\left(r-r_{1};t,t_{1};1-s\right)=q\left(t,1-s\right)$$

the structural factor can be expressed as:

(40) $$\begin{array}{ccc} S(ka;L/a) & = & \frac{1}{{\left(4\pi \right)}^{2}Q}\int_{0}^{1}ds\int_{0}^{s}ds^{\prime }\int dt^{\prime }dt[q\left(t^{\prime },s^{\prime }\right)G\left(k;t^{\prime };t,s-s^{\prime }\right)q^{*}\left(t,1-s\right)\\ & & \;\;\;\;\;\;+q\left(t,s^{\prime }\right)G\left(-k;t,t^{\prime };s-s^{\prime }\right)q^{*}\left(t^{\prime },1-s\right)] \end{array}$$

The structure factor S(ka;L/a,Γ) can be calculated through the integration of Equation (40). Its behaviors can be determined by two independent parameter L/a and Γ only.

Comparing to the structure factor without the orientational field as expressed by Equation (17), the structure factor of the ideal chain in the orientational field, Equation (40), involves two double integrals with respect to t(θ,ϕ) and $t^{\prime }\left(\theta^{\prime },\phi^{\prime }\right)$. It is a formidable task to compute and store the t and $t^{\prime }$ dependent propagators, because a sufficiently large degree of discretization is required in the orientational space to assure the accuracy of the multiple integral. This makes the difficulty in computing the structure factor in a nematic field. It should be noted that the propagator $G(k;t^{\prime };t,s-s^{\prime })$ includes two underlying *δ* functions, δ[t(s)−t] and $\delta [t\left(s^{\prime }\right)-t^{\prime }]$. The double integrals with respect to t and t in the expression structure factor assure the smooth constraint at the connecting joints between the propagators. Therefore, this can be performed analytically, and then, the numerical difficulty can be avoided. According to this consideration, the structure factor can be expressed by a formation similar to Equation (17).

(41) $$\begin{array}{ccc} S(ka;L/a) & = & 2\frac{1}{4\pi Q}\int_{0}^{1}ds_{1}\int_{0}^{s_{1}}ds_{2}\int dt\tilde{q}\left(t,s;k,s_{1},s_{2}\right) \end{array}$$

where the $\tilde{q}$ satisfies:

(42) $$\frac{\partial }{\partial s}\tilde{q}\left(t,s;k;s_{1},s_{2}\right)=\left[\frac{L}{a}\nabla_{t}^{2}+\eta \left(k,t,s_{1},s_{2}\right)+\Gamma \left(t_{z}^{2}-\frac{1}{3}\right)\right]\tilde{q}\left(t,s;k;s_{1},s_{2}\right)$$

where the external field depends on contour variable *s*:

(43) $$\begin{array}{c} \eta \left(k,t,s_{1},s_{2}\right)=\left\{\begin{array}{cc} iLk\cdot t & \mathrm{for}\;s\in [s_{1},s_{2}]\\ 0 & \mathrm{otherwise} \end{array}\right. \end{array}$$

and the initial condition is $\tilde{q}\left(t,s=0;k;s_{1},s_{2}\right)=1$.

Choose the direction of the nematic as the *z* axis and the direction of the projection of vector k in the *x*–*y* plane as the *x* axis. Thus, the structure factor of a WLC in a nematic phase is an anisotropic function depending on the orientation of the wavevector with respect to the nematic axis, $\theta_{k}$. By considering the expression:

(44) $$k\cdot t=k(\sin\theta_{k}\sin\theta \cos\varphi +\cos\theta_{k}\cos\theta )$$

the MDE becomes:

(45) $$\frac{\partial }{\partial s}\tilde{q}\left(\theta ,\varphi ,s;k,\theta_{k};s_{1},s_{2}\right)=\left[\frac{L}{a}\nabla_{t}^{2}+\eta \left(k,\theta_{k},\theta ,\varphi ,s_{1},s_{2}\right)+\Gamma \left(\cos^{2}\theta -\frac{1}{3}\right)\right]\tilde{q}\left(\theta ,\varphi ,s;k,\theta_{k};s_{1},s_{2}\right)$$

where:

(46) $$\begin{array}{c} \eta \left(k,\theta_{k},\theta ,\varphi ,s_{1},s_{2}\right)=\left\{\begin{array}{cc} iLk(\sin\theta_{k}\sin\theta \cos\phi +\cos\theta_{k}\cos\theta ) & \mathrm{for}\;s\in [s_{1},s_{2}]\\ 0 & \mathrm{otherwise} \end{array}\right. \end{array}$$

with initial condition, $\tilde{q}\left(\theta ,\varphi ,0;k,\theta_{k};s_{1},s_{2}\right)=1$. Given $k(k,\theta_{k})$, the pattern of the structure factor $S(k,\theta_{k})$ can be obtained by the double integration in Equation (41). Its integrand can be computed by solving Equation (45).

**Figure 2:**
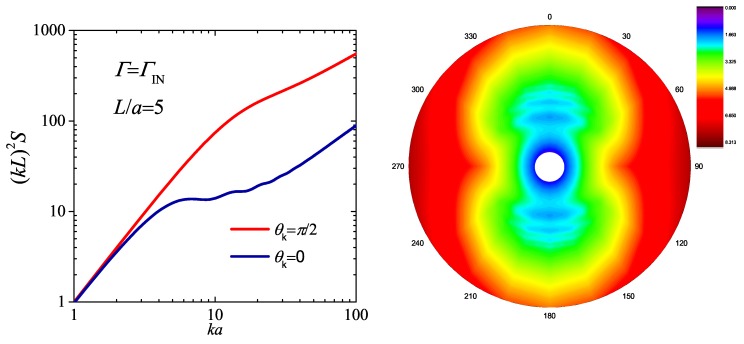
Structure factor, $S(\theta_{t},k)$, of the WLC for L/a=5 in a the nematic phase. The left panel shows the component of $\theta_{t}=0$ and π/2 in double-logarithmic Kratky plot. The right panel demonstrates the anisotropic properties of $S(\theta_{t},k)$ in a polar coordinate.

Taking the chain with L/a=5 in the nematic matrix as an example, its structure factors parallel to the nematic axis ($\theta_{k}=0$) and perpendicular to the nematic axis ($\theta_{k}=\pi /2$) are plotted in the left panel of Figure 2, and the whole structure factor $S(\theta_{t},k)$ is demonstrated in the right panel. In the small *k* region, both components satisfy the Guinier regime. In this condition, the chain is no longer isotropic, since different components do not coincide with each other. Its inertia tensor has two independent intrinsic values corresponding to $R_{g∥}$ and $R_{g⊥}$, respectively, which can be predicted by drawing the $S(\theta_{t}=0,k)$ and $S(\theta_{t}=\pi /2,k)$ in the Guinier plot. In order to give a comprehensive illustration of the anisotropic properties of the WLC in an orientational field, $S(\theta_{t},k)$ are plotted in a polar coordinate depending on $\theta_{t}$. The further effects of the orientational field on the conformation of semiflexible chain can be found in [63].

## 4. Gaussian Fluctuation Theory and the Random Phase Approximation

The structure factor of the ideal chain determined in the previous section is the starting point of Gaussian fluctuation theory. This theory is generally used to determine the structure factor of an order structure and then to analyze its stability. Its idea is considering the weak fluctuation around the reference state obtained form self-consistent field theory. Keeping secondary order approximation, the secondary term of the effective Hamiltonian is a matrix. Diagnosing this matrix, this term can be expressed as the summation of a set of harmonic oscillators. The moduli of these oscillators, which are the intrinsic values of the matrix, are the energy costs of the intrinsic fluctuation modes of the order structures. Moreover, due to this simple formation, its partition function can be obtained using the Gaussian integral, and then, the free energy can be predicted approximately. This method is called random phase approximation (RPA). This theory has been used to predict the structure factor of linear [22], comb, star [64] and miktoarm star [65] diblock copolymers. Furthermore, the dynamic structure factor has been predicted by Semenov et al. [66].

In this section, the Gaussian fluctuation theory of the WLC model will be demonstrated using the microphase separation in worm-like AB block copolymers as an example system. The WLC model can describe the statistics of the polymer chain in the entire L/a range. Therefore, the Gaussian fluctuation theory based on WLC is required for the comprehensive understanding of the phase behavior of the diblock polymer system [58]. Because of the difficulty of obtaining the exact structure factor of an ideal WLC, the Gaussian fluctuation theory had not been applied on the WLC system for the entire range of L/a until 2014. Many attempts based on the approximate structure factor of ideal WLC are made to predict the spinodal line of the semiflexible diblock copolymer. Approximating a semiflexible chain by freely-jointed rods of finite total length, Singh et al. provided a mechanism of constructing the correlation functions for such a chain [6]. In this model, the polymer conformation is controlled by the total number of monomers contained in the chain and the number of monomers in the rigid segment. As the ratio between these two numbers is large, the physical behavior is governed by the random walk configurations; otherwise, as the the ratio approaches unity, the physical behavior becomes rigid-rod alike. Coupling the expression with the RPA method, they analyzed the phase behavior of diblock copolymers based on this model in a number of scenarios, including the microphase separation of worm-like diblock copolymers from a homogeneous solution. One interesting conclusion made by these authors is the critical point where a system of homogenous symmetric AB diblock copolymers makes a phase transition to the microphase separated ordered structure; their estimation of the transition point for flexible chains gave ${\left(\chi L/a\right)}_{c}=10.5$, which matches that determined from a GSC model [22]. The transition point for symmetric rod-rod copolymers where the two blocks can freely rotate around the joint gave ${\left(\chi L/a\right)}_{c}=8.3$ [67]. Later work [68,69] predicts ${\left(\chi L/a\right)}_{c}=6.135$ for the symmetric rod-rod jointed by a straight line. Friedel and coworkers estimated the structural factor of a semiflexible-chain model in terms of a short cumulant expansion [70]. This approximation requires that the mean monomer-monomer distance $|R\left(s\right)-R\left(s^{\prime }\right)|$ is much smaller than the typical wavelength 2π/k; in such a circumstance, the local properties of the chain are totally ignored. Therefore, for the flexible-chain limit, this approximation can effectively recover the prediction of the Gaussian-chain model. However, for a polymer chain with finite rigidity, the approximation starts to suffer. Their prediction of ${\left(\chi L/a\right)}_{c}=8.4$ for the critical point of the disorder-order transition in symmetric rod-rod copolymers is above the value ${\left(\chi L/a\right)}_{c}=6.135$.

### 4.1. Gaussian Fluctuation Theory of the Worm-Like Diblock Copolymer

Consider an incompressible system of *n* monodisperse AB diblock copolymer chains, each with $N_{A}$ and $N_{B}$ monomers for the A block and the B block, respectively. The fractional composition of the polymer chains is given by $f=N_{A}/N$. For the sake of simplicity, each type of block is assumed to have the same Kuhn length *a* and equal monomer volume $\rho_{0}^{-1}$. The contour length for block copolymers denoted by *L* is related to the segment number $N=N_{A}+N_{B}$ by L=Na.

The local immiscibility can be described by the enthalpic penalty of mixing unlike segments in terms of the Flory–Huggins-type expression [16]. Instead of taking the point-like *δ* function adopted in most of the previous studies [14], we incorporate a generalizable function h(R) involving a finite range *ϵ* explicitly accounting for the volume effect of segments as:

(47) $$H_{1}=\chi \rho_{0}\int dr\int dr^{\prime }h(|r-r^{\prime }\left|\right){\hat{\phi }}_{A}\left(r\right){\hat{\phi }}_{B}\left(r^{\prime }\right)$$

The function h(R) is allowed to take any formalism as long as the normalization condition ∫dRh(R)=1 is satisfied. With the assumption of the same form h(R) adopted for all pairs, A–A, B–B and A–B, for convenience, the Flory–Huggins-like parameter *χ* manifests the net interaction arising from all components. Furthermore, the effective interaction range *ε* can be calculated by $\varepsilon^{2}=\int dR\;R^{2}h\left(R\right)$. Without loss of generality, two interaction potential functions with finite interaction are considered. First is a Gaussian distribution for the function:

(48) $$h\left(R\right)=\frac{1}{{\left(2\pi \right)}^{3/2}\varepsilon^{3}}\exp\left(-\frac{R^{2}}{2\varepsilon^{2}}\right)$$

Second is the Yukawa-type potential, which is typically used to characterize the screening effects on the pairwise interaction, such as in a polymer solution with high concentration and in a polyelectrolyte system. It has the form of:

(49) $$h_{Y}\left(r\right)=\frac{1}{4\pi \varepsilon^{2}r}\exp\left(-\frac{r}{\varepsilon }\right)$$

characterizing the screen effect with a finite screen length *ε*. The density operators for the each component is defined respectively as:

(50) $${\hat{\phi }}_{A}\left(r\right)\equiv \frac{N}{\rho_{0}}\sum_{k\;=\;1}^{n}\int_{0}^{f}ds\delta \left[r-R_{k}\left(s\right)\right]$$

(51) $${\hat{\phi }}_{B}\left(r\right)\equiv \frac{N}{\rho_{0}}\sum_{k\;=\;1}^{n}\int_{f}^{1}ds\delta \left[r-R_{k}\left(s\right)\right]$$

The effective Hamiltonian can be expressed by:

(52) $$\begin{array}{cc} \beta F=-\ln Q+\frac{1}{V}\int dr & \{\chi N\int dr^{\prime }h(|r-r^{\prime }\left|\right)\phi_{A}\left(r\right)\phi_{B}\left(r^{\prime }\right)-w_{A}\left(r\right)\phi_{A}\left(r\right)-w_{B}\left(r\right)\phi_{B}\left(r\right)\\ & +\xi \left(r\right)\left[\phi_{A}\left(r\right)+\phi_{B}\left(r\right)-1\right]\} \end{array}$$

It is a functional of the mean fields $w_{A}\left(r\right)$ and $w_{B}\left(r\right)$ that the components experience, the mean volume fraction distributions $\phi_{A}\left(r\right)$ and $\phi_{B}\left(r\right)$ at coordinate r and a Lagrangian multiplier ξ(r) that enforces the incompressibility constraint on the system. The reduced parameter χN denotes the effective interaction between different blocks. The function *Q* called the single chain partition function can be calculated from:

(53) $$Q=\frac{1}{4\pi V}\int drdtq\left(r,t,1\right)$$

The propagator q(r,t,s) can be obtained by solving the MDE: [14,33]

(54) $$\frac{\partial }{\partial s}q\left(r,t,s\right)=\left[N\nabla_{t}^{2}-Lt\cdot \nabla_{r}-w\left(r,t,s\right)\right]q\left(r,t,s\right)$$

subject to the initial condition q(r,t,0)=1. For the current system,

$$w\left(r,t,s\right)=\left\{\begin{array}{cc} w_{A}\left(r,t\right), & \mathrm{if}0\leq s\leq f\\ w_{B}\left(r,t\right), & \mathrm{if}f<s\leq 1 \end{array}\right.$$

A second segment distribution function $q^{*}\left(r,t,s\right)$ complementary to q(r,t,s) is needed, due to the distinct ends of the diblock copolymers. It satisfies a similar MDE:

(55) $$\frac{\partial }{\partial s}q^{*}\left(r,t,s\right)=\left[-N\nabla_{t}^{2}-Lt\cdot \nabla_{r}+w\left(r,s\right)\right]q^{*}\left(r,t,s\right)$$

and is subject to the initial condition $q^{*}\left(r,t,1\right)=1$.

The self-consistent field theory equations can be obtained by minimizing the free energy functional in Equation (52) with respect to the functions δH/δζ=0, where $\zeta =\phi_{A}$, $\phi_{B}$, *ξ*, $w_{A}$ and $w_{B}$,

(56) $$w_{A}\left(r\right)=\chi N\int dRh\left(R\right)\phi_{B}(|r-R|)+\xi \left(r\right)$$

(57) $$w_{B}\left(r\right)=\chi N\int dRh\left(R\right)\phi_{A}(|r-R|)+\xi \left(r\right)$$

(58) $$\phi_{A}\left(r\right)+\phi_{B}\left(r\right)=1$$

(59) $$\phi_{A}\left(r\right)=\frac{1}{4\pi Q}\int dt\int_{0}^{f}dsq\left(r,t,s\right)q^{*}\left(r,t,s\right)$$

and:

(60) $$\phi_{B}\left(r\right)=\frac{1}{4\pi Q}\int dt\int_{f}^{1}dsq\left(r,t,s\right)q^{*}\left(r,t,s\right)$$

The solution of these equations, $\zeta^{*}\left(r\right)$, represents the equilibrium field configurations in the mean-field level. Filling $\zeta^{*}\left(r\right)$ back into Equation (52), we can obtain $F^{*}=H^{*}$, the mean-field free energy determined at the mean-field level.

The solution for the spatially-homogenous phase is r-independent. In order to examine the stability of the homogeneous phase, one considers the weak inhomogeneous condition; the self-consistent field theory (SCFT) equations can be solved by the random phase approximation (RPA). This approach is based on the linear analysis of the structure, which can determine the spinodal line and the most unstable fluctuation mode. Considering the fluctuations, δζ(r), around the mean-field solution $\zeta^{*}$,

(61) $$\zeta \left(r\right)=\zeta^{*}+\delta \zeta \left(r\right)$$

The effective Hamiltonian can then be expressed as $\beta F\approx \beta F^{*}+\beta F^{\left(2\right)}$, where $\beta F^{\left(2\right)}$ is the Gaussian fluctuation contribution [23]. The partition function can be approximated by:

(62) $$\begin{array}{ccc} Z & \approx & \exp\left(-\beta F^{*}\right)\int D\left\{\delta \phi \right\}\exp\left\{-\beta F^{\left(2\right)}\left[\delta \phi \right]\right\} \end{array}$$

where $\delta \phi \equiv \delta \phi_{A}-\delta \phi_{B}$, and in the k space,

(63) $$\begin{array}{c} \beta F^{\left(2\right)}=\frac{1}{2}\int \frac{dk}{{\left(2\pi \right)}^{3}}\delta \phi \left(k\right)C_{\mathrm{RPA}}^{-1}\left(k\right)\delta \phi \left(-k\right) \end{array}$$

$C_{\mathrm{RPA}}\left(k\right)$ is the k space representation of the so-called RPA correlation function. It is the effective pairwise interaction between monomers. Its inverse is the structure factor of the multi-chain system, which characterizes the modulus of its corresponding fluctuation mode, δϕ(k). Because the spacial homogeneous solution of the SCFT is considered as the reference state, the correlation function only depends on the modulus of the wavevector, *k*. It can be expressed in terms of the intra-chain correlation functions $C_{AA}$, $C_{BB}$ and $C_{AB}$,

(64) $$\begin{array}{c} C_{RPA}^{-1}\left(k\right)\equiv \frac{C_{AA}\left(k\right)+2C_{AB}\left(k\right)+C_{BB}\left(k\right)}{4\left[C_{AA}\left(k\right)C_{BB}\left(k\right)-C_{AB}^{2}\left(k\right)\right]}-\frac{1}{2}\chi N\tilde{h}\left(k\right) \end{array}$$

The intra-chain correlation functions can be computed by the method discussed in the previous section,

(65) $$\begin{array}{ccc} C_{AA}\left(k\right) & = & \frac{1}{4\pi }\int_{0}^{f}ds\int_{0}^{s}ds^{\prime }dt\left[G\left(k,t,s-s^{\prime }\right)+G\left(-k,t,s-s^{\prime }\right)\right],\\ C_{BB}\left(k\right) & = & \frac{1}{4\pi }\int_{0}^{1-f}ds\int_{0}^{s}ds^{\prime }dt\left[G\left(k,t,s-s^{\prime }\right)+G\left(-k,t,s-s^{\prime }\right)\right],\\ C_{AB}\left(k\right) & = & C_{BA}\left(k\right)=\left[S\left(k\right)-C_{AA}\left(k\right)-C_{BB}\left(k\right)\right]/2 \end{array}$$

In Equation (65), $\tilde{h}\left(k\right)$ is the potential function in reciprocal space. For the Gaussian formation,

(66) $$\begin{array}{c} {\tilde{h}}_{G}\left(k\right)=\exp\left(-\frac{k^{2}\varepsilon^{2}}{2}\right) \end{array}$$

For the Yukawa formation,

(67) $$\begin{array}{c} {\tilde{h}}_{Y}\left(k\right)=\frac{1}{k^{2}\varepsilon^{2}+1} \end{array}$$

Both types of potentials will be compared in the RPA approximation computation. $C_{\mathrm{RPA}}^{-1}\left(k\right)$ can be considered as the modulus of the fluctuation mode δϕ(k). Once $C_{\mathrm{RPA}}^{-1}\left(k\right)$ is determined, we search for the minimum of this function numerically, which determines the soft mode $k^{*}$. As $C_{\mathrm{RPA}}^{-1}\left(k^{*}\right)$ is decreased to zero, the homogenous phase loses its stability, and the spinodal point can be arrived at by:

(68) $$\begin{array}{c} {\left(\chi N\right)}_{s}=\min\left[\tilde{C}\left(k\right)/\tilde{h}\left(k\right)\right] \end{array}$$

where:

(69) $$\begin{array}{c} \tilde{C}\left(k\right)\equiv \frac{C_{AA}\left(k\right)+2C_{AB}\left(k\right)+C_{BB}\left(k\right)}{2\left[C_{AA}\left(k\right)C_{BB}\left(k\right)-C_{AB}^{2}\left(k\right)\right]} \end{array}$$

The domain size of the order structure formed by micro-phase separation can be determined by $D\equiv 2\pi /k^{*}$.

### 4.2. Worm-Like Diblock Polymer with *δ* Interaction Potential

**Figure 3:**
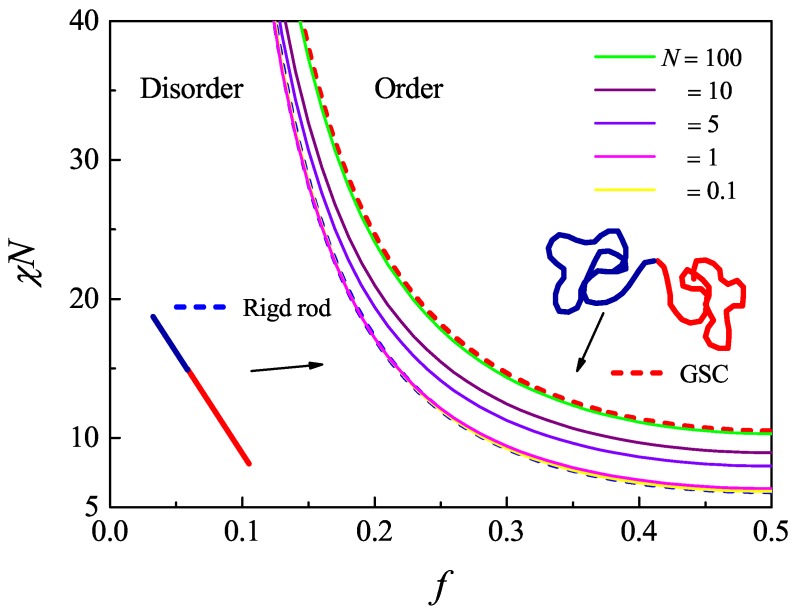
Spinodal lines for a worm-like diblock copolymer melt for various chain flexibilities, L/a=0.1, 1, 5, 10 and 100, are shown. The curves are within the region bounded by an upper solid red curve and a lower dashed blue curve, produced from the analytic expressions for the structure factors of a Gaussian-chain model and a rigid-rod model, respectively.

The numerical results of the spinodal curve for diblock polymers is presented in Figure 3 for systems that have various degrees of flexibility L/a. In the current case, the phase diagram in the entire range of the *A* volume fraction f=[0,1] is symmetric with respect to f=0.5. Only half of the region f=[0,0.5] is plotted in the figure. For comparison, the spinodal lines computed based on the Gaussian chain model and rigid rod chain model, by directly using the analytic expressions in Equations (4) and (23), respectively, are produced and plotted in the figure as the red and blue dashed lines.

As the flexibility L/a increases, the WLC spinodal curve approaches the Gaussian-chain spinodal curve. It takes as high as L/a=100 to closely reach the red dashed curve in Figure 3. Lowering of the flexibility helps the expansion of the ordered phase region. This can be qualitatively explained by the consideration of the effects of the conformational entropy of a WLC. In the ordered state, the Flory–Huggins repulsion between the *A* and *B*blocks dominates and drives the phase separation. The polymer chains are then stretched accompanied by a reduction of the chain conformational entropy. Under otherwise the same physical conditions, a more rigid chain is always more extended than a more flexible chain; hence, the entropic loss of a rigid chain in forming an extended conformation in an ordered state is less than that of a flexible chain; accordingly, the ordered region expands. As the flexibility L/a further decreases, the spinodal curve eventually approaches the prediction obtained based on the rigid-rod model, the dashed blue curve in Figure 3. The curve for the case of L/a=0.1 already reaches the rigid rod limit significantly.

**Figure 4:**
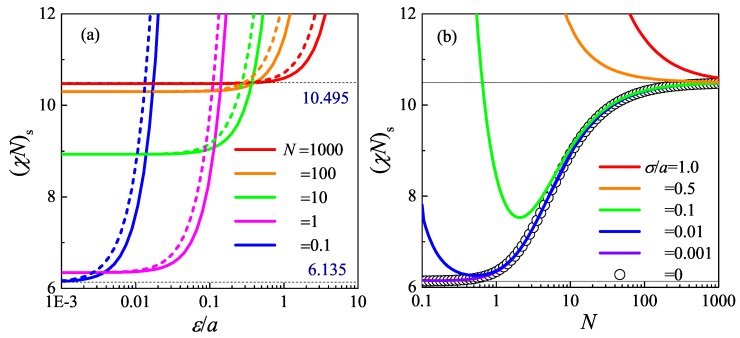
Effects of the interaction range on the spinodal curve of a worm-like diblock copolymer as functions of the interaction range ϵ/a and *N* [73]. In (**a**), intermediate cases for various *N* are shown, between the flexible (large-*N*) and rod-like (small-*N*) limits. Solid and long-dashed curves represent the numerical results of the Gaussian and Yukawa interaction functions, respectively. In (**b**), various cases for interaction ranges as indicated are shown.

For a fixed f=1/2, the spinodal points as a function of L/a are represented as circle symbols in Figure 4b. This is a case where the system undergoes a second-order phase transition from the disordered state to the lamella state, as has been discussed previously in [68,69]. It is this special case where the spinonal curve coincides with the second-order transition curve. Within the Gaussian chain model, the order-disorder-transition critical point for a symmetric diblock copolymer melt occurs at χN=10.495, which is a well-determined value in the literature [22]. As the flexibility L/a is lowered, the critical point χN decreases as illustrated as circle symbols in Figure 4b. When L/a≪1, the polymer chain becomes rigid, and the spinodal curve approaches χL/a=6.1352, in the rigid-rod limit. Both limits are represented as dots in Figure 4. The spinodal curve ${\left(\chi N\right)}_{s}\left(L/a\right)$ for f=0.5 predicted by Gaussian fluctuation theory fully agrees with that of the binodal curve determined by SCFT [68,69]. This indicates the in the mean-field theory level, f=0.5 is a critical point for the entire range of L/a. However, a further study on the rod-coil diblock copolymer spinodal curve and binodal curve shows theynever intersect [71]. This means there is not any critical point in the rigidly-asymmetric block copolymer. In the intermediate L/a∈(1,100) range, the spinodal curve makes a sharp change. Within this region, the statistical property of a worm-like chain is of a typical semiflexible nature.

### 4.3. Effects of the Finite Interaction Range

For short block copolymers, the size of the narrowing interfacial width is much less than the size for long block copolymers, even less than one statistic Kuhn length [72]. Then, one anticipates that the volume of the coarse-grained segment, which usually consists of several tens of monomers [14], will play an extremely significant role in the phase separation for short block copolymers. The influence of segmental volume is via the manner of the distance-dependent interactions between segments on the stability of phase transitions of diblock copolymer melts.

In the field theory of polymeric systems, the coarse-grained *δ* function pair interaction potential and Gaussian chain model are widely used. In this approximation, the properties on the length scale of interaction range, *ε*, and the Kuhn length, *a*, are ignored. This is because the characteristic length scale of the long chain systems in a weak inhomogeneous condition is around the radius of gyration of polymer chains, $R_{g}$. It is in the mesoscopic length scale and much larger than the microscopic length scale of interaction range *ε* and Kuhn length *a*. According to the idea of renormalization, all of the properties in the microscopic length scale are not important to the phase behaviors and can be ignored. Therefore, the Gaussian chain model is a good approximation to characterize the statistic properties of polymer in the mesoscopic length scale. Accordingly, the *δ* function potential is sufficient for the theory using Gaussian chain model. Based on these coarse-grained models, the solution of the field theory is greatly simplified, and the theory is widely used to study the phase behaviors of the polymeric system. However, the phase behaviors of many polymeric systems involve multi-length scales. The WLC model includes two length scales, the persistence length and the chain length. It extends the field theory of the polymeric system to the condition of the chain with a small length and the condition of the long chain in the fully stretched state. In these conditions, the characteristic length is much less than the chain length, and the properties in the microscopic length scale become important. Because the WLC model can resolve the length scale down to the Kuhn length, the interaction model should be consistent with the resolution of the WLC model accordingly. However, the interaction range effects have not been considered in the theory based on the WLC model before. The quantitative verification of the coarse-grained approximation of the interaction potential is required. The validity of the *δ* function potential is checked by solving the WLC model incorporating the potential function with a finite explicit interaction range.

According to the RPA shown in Equation (64), the spinodal point, ${\left(\chi N\right)}_{s}$, depends on the formulation of potential function $\tilde{h}\left(k\right)$, which has a characteristic length *ε*. For the limit of ε→0, $\tilde{h}\left(k\right)\to 1$ for both types of potential functions. Additionally, then, the spinodal point recovers to the predication of the theory with the *δ* function type interaction, which can be determined by:

(70) $$\begin{array}{c} {\left(\chi N\right)}_{0}=\min\left[\tilde{C}\left(k\right)\right] \end{array}$$

For the condition that a finite *ε* is considered, both the Gaussian-type potential and the Yukawa-type potential have similar behaviors on the limits of both small and large length scales. For the length scale much larger than *ε*, i.e, k→0), $\tilde{h}\left(k\right)\to 1$. The modulus of fluctuation δϕ(k=0) has the same behavior of the system with *δ* function potential. For the length scale much less than *ε*, i.e., k→∞, $\tilde{h}\left(k\right)$ approaches zero. It increases the modulus of fluctuation mode ϕ(k→∞) dramatically, which stabilizes the system with respect to the fluctuation with a wavelength smaller than the interaction range *ε*, and it is hard to form a structure with a characteristic length much smaller than *ε* in this system. For the fluctuation mode δϕ with wavelength *k* around *ε*, the finite interaction range always increases the modulus of the fluctuation and then increases the spinodal point ${\left(\chi N\right)}_{s}$ of the system.

Both the Gaussian-type potential and the Yukawa-type potential incorporating with the WLC model are considered in the RPA computation to investigate the effect of the interaction range on the phase behavior. The spinodal points of the block copolymer with f=0.5 as a function of interaction range ε/a for different *N* are compared in Figure 4a. For the limit of ε=0, ${\left(\chi N\right)}_{s}$ increases from 6.135–10.495, as *N* changes from 0–*∞* monotonically. Because the finite interaction range tends to stabilize the fluctuation except δϕ(k=0), the spinodal point ${\left(\chi N\right)}_{s}$ of the system with finite *ε* always is larger than that of the system with ε=0. As indicated by Figure 4a, the spinodal points increases, as ε/a increases in a two-step process. A threshold of interaction range $\varepsilon_{c}$ can be determined for spinodal lines of all *N*. For $\varepsilon <\varepsilon_{c}$, the spinodal point is nearly independent of *ε*. On the contrary, for $\varepsilon >\varepsilon_{c}$, the spinodal point increases rapidly as *ε* increases. In addition, the results of different interaction potential formulations are distinct from each other. Here, the spinodal point of the system with the Yukawa-type potential is higher than that with the Gaussian-type potential. The deviation is up to 30%. These results indicate that $\varepsilon_{c}$ serves as a criterion length of coarse-graining, which indicates the resolution limit of the theory. The effect of the interaction range can be ignored for $\varepsilon <\varepsilon_{c}$. For large *N*, e.g., N=1000, $\varepsilon_{c}/a∼1$. When interaction range *ε* is smaller than the Kuhn length *a*, ${\left(\chi N\right)}_{s}\approx 10.495$, which almost does not depend on the microscopic details, the interaction range and the formulation of the potential. In this condition, the effects of these microscopic properties of the system can be ignored, and the theory based on the Gaussian chain model and the *δ* function interaction is a good approximation. While for *ε* is around or larger than *a*, the microscopic details become important, and the theory based on the *δ* function interaction potential is not sufficient to characterize the phase behavior of the polymeric system. The potential function with finite interaction range becomes necessary. The $\varepsilon_{c}$ depends on the chain length, *N*. As *N* decreases, $\varepsilon_{c}/a$ decreases continuously, which means the criterion that the interaction potential can be approximated by the *δ* function potential decreases. For small *N*, e.g., N=0.1, $\varepsilon_{c}/a$ is less than $10^{-3}$, which means for the chain with low molecular weight the microscopic details of the interaction cannot be ignored. A tiny change of the interaction potential formulation or interaction range leads to a dramatic deviation of the spinodal point. It can be concluded from Figure 4a that the *δ* function interaction is a good coarse-grained approximation for the system only for the system with large *N*. For the low molecular weight system, the interaction range affects the spinodal point seriously.

To demonstrate the *N* dependence of the effect of the interaction range, the spinodal point, ${\left(\chi N\right)}_{s}$, and the domain size, D/a, of the order structure formed by the phase separation are summarized in Figure 4. For the limit of ε/a=0 (circles in Figure 4b), ${\left(\chi N\right)}_{s}$ increases monotonically as *N* increases for N∈(1,00), while it almost does not change with *N* for both small and large *N*. This behavior is from the increase of conformational entropy as *N* increases [58]. Once a finite interaction range *ε* is considered in the model, the interaction range dramatically increases the spinodal point of the system with small and medium *N*. As *N* increases, the effect of *ε* diminishes and at a finite $N_{c}$; the spinodal point recovers to the result of ε=0. For $N>N_{c}$, the effects of the interaction range can be ignored. *N* has contradictory contributions to the entropy effect and interaction range effect. For small *ε* where $N_{c}<100$, these contradictory contributions make the function of ${\left(\chi N\right)}_{s}$ vs. *N* become concave, and a minimum spinodal point appears. As *ε* increases, the effect of the iteration range is enhanced, and $N_{c}$ increases continuously. For the condition with a sufficiently large *ε*, $N_{c}>100$, and then, ${\left(\chi N\right)}_{s}$ becomes a monotonic decreasing function of *N*. Basically, the effects of the interaction range are from the competition between the characteristic length of the phase separation, D/a, and the interaction range, ε/a. As mentioned above, $\tilde{v}\left(k\right)\to 0$ for *k* larger than 2π/ε, which makes the modulus of the fluctuation with high frequency increase rapidly and prevents the micro-phase separation. To make the fluctuation δϕ(k>2π/ε) unstable, it will take much higher χN. This is from the excluded volume interaction to prevent the overlapping between monomers. Therefore, the interaction range *ε* serves as a length scale cutoff. No structure with a length scale smaller than ε/a can be formed.

## 5. Summary

In summary, the recent development on the prediction of the structure factor of the WLC model and its application in Gaussian fluctuation theory are reviewed. The WLC model is a typical multiscale chain model, which can describe the statistic properties of the polymer chain from the Kuhn length, *a*, to the chain length, *L*. It is a natural request to use the WLC model instead of the GSC model when the system characteristic length scale is much lower than $R_{g}$. Besides the spacial degree of freedom, the WLC model also includes the orientational degree of freedom. Therefore, studying the anisotropic system, such as the liquid crystal WLC model, is required. Although the WLC model has been put forward for many decades, the structure factor of WLC was not determined exactly for the entire parameter space of L/a and ka until 2004 [54,55]. More recently, the numerical method based on direct solving of the MDE in *k* space has been reported, which can be easily extended to the condition of WLC in the external field. As a consequence, this method is useful for the Gaussian fluctuation field theory in which the intra-chain coordination function is required. On the basis of the Gaussian fluctuation, another length scale, i.e., the interaction potential range, which characterizes the thickness of the chain can be studied in the framework of the WLC system. A number of other important polymer problems can be examined using the current numerical method for WLCs, such as the fluctuation effect by incorporating with the Hartree approximation and the correlation effect by introducing the liquid state theory. Moreover, the RPA correlation function is the linear response function of the system to the external stimulation. Therefore, Gaussian fluctuation theory can be used to predict the properties from the structure of the material. More attention should be paid to the Gaussian fluctuation theory of the WLC system for both functional material science and biological science.

## Figures and Tables

**Table 1:** Parameters used in Pedersen’s expression of the structure factor of the WLC model for both rigid and flexible conditions.

	$b_{1}$	$b_{2}$	$P_{1}$	$P_{2}$
L/a>2	1	1	5.33	5.53
L/a≤2	0.0625	0	3.95	11.7a/L

## Abbreviations

The following abbreviations are used in this manuscript:

GSC: Gaussian chain
MDE: modified diffusion equation
RPA: random phase approximation
SCFT: self-consistent field theory
WLC: worm-like chain

## References

[1] Netz R.R., Orland H. (1999). Variational theory for a single polyelectrolyte chain. Eur. Phys. J. B.

[2] Bates C.M., Maher M.J., Janes D.W., Ellison C.J., Willson C.G. (2014). Block copolymer lithography. Macromolecules.

[3] Schmid F., Schick M. (1995). Liquid phases of Langmuir monolayers. J. Chem. Phys..

[4] Katsov K., Müller M., Schick M. (2004). Field theoretic study of bilayer membrane fusion. I. Hemifusion mechanism. Biophys. J..

[5] Tanner P., Baumann P., Enea R., Onaca O., Palivan C., Meier W. (2011). Polymeric vesicles: From drug carriers to nanoreactors and artificial organelles. Acc. Chem. Res..

[6] Singh C., Goulian M., Liu A., Fredrickson G.H. (1994). Phase behavior of semiflexible diblock copolymers. Macromolecules.

[7] Marques C. M., Fredrickson G. H. (1997). Rigid gaussian chains I: The scattering function. J. Phys. II Fronce.

[8] Kratky O., Porod G. (1949). Röntgenuntersuchung glöster Fagenmoleküle. Recl. Trav. Chim..

[9] Saito N., Takahashi K., Yunoki Y. (1967). The statistical mechanical theory of stiff chains. J. Phys. Soc. Jpn..

[10] Liu A.J., Fredrickson G.H. (1993). Free energy functionals for liquid crystalline polymer solutions and blends. Macromolecules.

[11] Cui S.M., Akcakir O., Chen Z.Y. (1995). Isotropic-nematic interface of liquid-crystalline polymers. Phys. Rev. E.

[12] Düchs D., Sullivan D.E. (2002). Entropy-induced smectic phases in rod-coil copolymers. J. Phys. Condens. Matter.

[13] Jiang Y., Chen Z.Y. (2010). Isotropic-nematic interface in a lyotropic system of worm-like chains with the Onsager interaction. Macromolecules.

[14] Fredrickson G.H. (2006). The Equilibrium Theory of Inhomogeneous Polymers.

[15] Higgins J.S., Benoit H.C. (1994). Polymers and Neutron Scattering.

[16] Flory P.J. (1953). Principles of Polymer Chemistry.

[17] Doi M., Edwards S.F. (1986). The Theory of Polymer Dynamics.

[18] Benoit H., Doty P. (1953). Light scattering from non-gaussian chains. J. Phys. Chem..

[19] Hermans J.J., Ullman R. (1952). The statistics of stiff chains, with applications to light scattering. Physica.

[20] Hammouda B., Doty P. (1993). SANS from Homogeneous polymer mixtures: A unified overview. Adv. Polym. Sci..

[21] Yeung C., Shi A.C., Noolandi J., Desai R.C. (1996). Anisotropic fluctuations in ordered copolymer phases. Macromol. Theory Simul..

[22] Leibler L. (1980). Theory of microphase separation in block copolymers. Macromolecules.

[23] Shi A.-C., Noolandi J., Desai R.D. (1996). Theory of anisotropic fluctuations in ordered block copolymer phases. Macromolecules.

[24] Fredrickson G.H., Helfand E. (1987). Fluctuation effects in the theory of microphase separation in block copolymers. J. Phy. Chem..

[25] Schweizer K.S., Curro J.G. (1997). Integral equation theories of the structure, thermodynamics, and phase transitions of polymer fluids. Adv. Chem. Phys..

[26] Netz R.R., Andelman D. (2003). Neutral and charged polymers at interfaces. Phys. Rep..

[27] Semenov A.N. (2002). Adsorption of a semiflexible worm-like chain. Eur. Phys. J. E.

[28] Nakamura I., Shi A.C., Wang Z.G. (2012). Ion solvation in liquid mixtures: Effects of solvent reorganization. Phys. Rev. Lett..

[29] Sing C.E., Zwanikken J.W., de la Cruz M.O. (2014). Electrostatic control of block copolymer morphology. Nat. Mater..

[30] Ariel G., Andelman D. (2003). Polyelectrolyte persistence length: Attractive effect of counterion correlations and fluctuations. Europhys. Lett..

[31] Chen K., Saltzman J., Schweizer K.S. (2010). Molecular theories of segmental dynamics and mechanical response in deeply supercooled polymer melts and glasses. Annu. Rev. Condens. Matter Phys..

[32] Dell Z.E., Schweizer K.S. (2013). Theory of localization and activated hopping of nanoparticles in cross-linked networks and entangled polymer melts. Macromolecules.

[33] Freed K.F. (1972). Functional integrals and polymer statistics. Adv. Chem. Phys..

[34] Dogic Z., Zhang J., Lau A., Aranda-Espinoza H., Dalhaimer P., Discher D.E., Janmey P.A., Kamien R.D., Lubensky T.C., Yodh A.G. (2004). Elongation and fluctuations of semiflexible polymers in a nematic solvent. Phys. Rev. Lett..

[35] Neugebauer T. (1943). Berechnung der lichtzerstreuung von fadenkettenlösungen. Ann. Phys..

[36] Teraoka I. (2002). Polymer Solutions: An Introduction to Physical Properties.

[37] Feigin L.A., Svergun D.I. (1987). Structure Analysis by Small-Angle X-ray and Neutron Scattering.

[38] Norisuye T., Fujita H. (1982). Excluded-volum effects in dilute polymer solutions. XIII. Efffects of chain stiffness. Polym. J..

[39] Hsu H.-P., Paul W., Binder K. (2010). Polymer chain stiffness vs. excluded volume: A Monte Carlo study of the crossover towards the worm-like chain model. Europhys. Lett..

[40] Hsu H.-P., Paul W., Binder K. (2011). Understanding the multiple length scales describing the structure of bottle-brush polymers by Monte Carlo simulation methods. Macromol. Theory Simul..

[41] Hsu H.-P., Paul W., Binder K. (2013). Estimation of persistence lengths of semiflexible polymers: Insight from simulations. Polym. Sci. Ser. C.

[42] Hsu H.-P., Binder K. (2012). Stretching semiflexible polymer chains: Evidence for the importance of excluded volume effects from Monte Carlo simulation. J. Chem. Phys..

[43] Kholodenko A.L. (1990). Fermi-bose transmutation: From semiflexible polymers to superstrings. Ann. Phys..

[44] Kholodenko A.L. (1992). Persistence length and related conformational properties of semiflexible polymers from Dirac propagator. J. Chem. Phys..

[45] Kholodenko A.L. (1993). Analytical calculation of the scattering function for polymers of arbitrary flexibility using the Dirac propagator. Macromolecules.

[46] Bawendi M.G., Freed K.F. (1985). A wiener integral model for stiff polymer chains. J. Chem. Phys..

[47] Ghosh K., Muthukumar M. (2001). Scattering properties of a single semiflexible polyelectrolyte. J. Polym. Sci. Part B Polym. Phys..

[48] Yoshizaki T., Yamakawa H. (1980). Scattering functions of worm-like and helical worm-like chains. Macromolecules.

[49] Pedersen J.S., Schurtenberger P. (1996). Scattering functions of semiflexible polymers with and without excluded volume effects. Macromolecules.

[50] FISH. http://www.diamond.ac.uk/Home/Beamlines/small-angle/SAXS-Software/CCP13/FISH/Models.html.

[51] SASFIT. http://sasfit.ingobressler.net/manual/KholodenkoWorm.

[52] Yamakawa H. (1997). Worm-Like Chains in Polymer Solutions.

[53] Stepanow S., Schütz G.M. (2002). The distribution function of a semiflexible polymer and random walks with constraints. Europhys. Lett..

[54] Stepanow S. (2004). Statistical mechanics of semiflexible polymers. Eur. Phys. J. B.

[55] Spakowitz A.J., Wang Z.-G. (2004). Exact results for a semiflexible polymer chain in an aligning field. Macromolecules.

[56] Mehraeen S., Sudhanshu B., Koslover E.F., Spakowitz A.J. (2008). End-to-end distribution for a worm-like chain in arbitrary dimensions. Phys. Rev. E.

[57] Tsubouchi R., Ida D., Yoshizaki T., Yamakawa H. (2014). Scattering function of worm-like rings. Macromolecules.

[58] Zhang X.H., Jiang Y., Miao B., Chen Y.L., Yan D.D., Chen J.Z.Y. (2014). The structure factor of a worm-like chain and the random-phase-approximation solution for the spinodal line of a diblock copolymer melt. Soft Matter.

[59] Deng M., Jiang Y., Liang H., Chen J.Z.Y. (2010). Worm-like polymer brush: A self-consistent field treatment. Macromolecules.

[60] Fujii M., Yamakawa H. (1977). Statistical mechanics of helical worm-like chains. III. Scattering functions. J. Chem. Phys..

[61] Maier W., Saupe A. (1958). Eine einfache molekulare Theorie des nematischen kristallinflüssigen Zustandes. Z. Naturforsch. A.

[62] Maier W., Saupe A. (1959). Eine einfache molekular-statistische Theorie der nematischen kristallinflüssigen Phase. Z. Naturforsch. A.

[63] Jiang Y., Zhang X.H., Miao B., Yan D.D. (2015). The study of the structure factor of a worm-like chain in an orientational external field. J. Chem. Phys..

[64] Benoit H., Hadziioannou G. (1988). Scattering theory and properties of block copolymers with various architectures in the homogeneous bulk state. Macromolecules.

[65] Zhang X.H., Chen Y., Qu L.J., Yan D.D. (2013). Effects of attractive colloids on the phase separation behaviors of binary polymer blends. J. Chem. Phys..

[66] Semenov A.N., Anastasiadis S.H., Boudenne N., Fytas G., Xenidou M., Hadjichristidis N. (1997). Dynamic structure factor of diblock copolymers in the ordering regime. Macromolecules.

[67] Borsali R., Lecommandoux S., Pecora R., Benoit H. (2001). Scattering Properties of rod-coil and once-broken rod block copolymers. Macromolecules.

[68] Matsen M.W. (1996). Melts of semiflexible diblock copolymer. J. Chem. Phys..

[69] Jiang Y., Zhang W.Y., Chen J.Z.Y. (2011). Dependence of the disorder-lamellar stability boundary of a melt of asymmetric worm-like AB diblock copolymers on the chain rigidity. Phys. Rev. E.

[70] Friedel P., John A., Pospiech D., Jehnichen D., Netz R.R. (2002). Modelling of the phase separation behaviour of semiflexible diblock copolymers. Macromol. Theory Simul..

[71] Tang J.Z., Jiang Y., Zhang X.H., Yan D.D., Chen J.Z.Y. (2015). Phase diagram of rod-coil diblock copolymer melts. Macromolecules.

[72] Matsen M.W. (2012). Self-consistent field theory for melts of low-molecular-weight diblock copolymer. Macromolecules.

[73] Jiang Y., Zhang X.H., Miao B., Yan D.D., Chen J.Z.Y. (2016). Microphase separation of short worm-like diblock copolymers with finite interaction range. Soft Matter.

